# Antithymocyte Globulin Induces a Tolerogenic Phenotype in Human Dendritic Cells

**DOI:** 10.3390/ijms17122081

**Published:** 2016-12-11

**Authors:** Tobias Roider, Michael Katzfuß, Carina Matos, Katrin Singer, Kathrin Renner, Peter J. Oefner, Katja Dettmer-Wilde, Wolfgang Herr, Ernst Holler, Marina Kreutz, Katrin Peter

**Affiliations:** 1 Department of Internal Medicine III, University Hospital Regensburg, Franz-Josef-Strauß-Allee 11, 93053 Regensburg, Germany; roidertobias@gmail.com (T.R.); michael.katzfuss@puk.zh.ch (M.K.); carina.matos@ukr.de (C.M.); katrin.singer@ukr.de (K.S.); kathrin.renner-sattler@ukr.de (K.R.); wolfgang.herr@ukr.de (W.H.); ernst.holler@ukr.de (E.H.); marina.kreutz@ukr.de (M.K.); 2 Institute of Functional Genomics, University of Regensburg, Am BioPark 9, 93053 Regensburg, Germany; peter.oefner@ukr.de (P.J.O.); katja.dettmer@ukr.de (K.D.-W.)

**Keywords:** allogeneic hematopoietic stem cell transplantation, dendritic cell, antithymocyte globulin, Grafalon, tolerogenic, indoleamine 2,3-dioxygenase, immunosuppressive

## Abstract

Antithymocyte globulin (ATG) is used in the prevention of graft-versus-host disease during allogeneic hematopoietic stem cell transplantation. It is generally accepted that ATG mediates its immunosuppressive effect primarily via depletion of T cells. Here, we analyzed the impact of ATG-Fresenius (now Grafalon^®^) on human monocyte-derived dendritic cells (DC). ATG induced a semi-mature phenotype in DC with significantly reduced expression of CD14, increased expression of HLA-DR, and intermediate expression of CD54, CD80, CD83, and CD86. ATG-DC showed an increase in IL-10 secretion but no IL-12 production. In line with this tolerogenic phenotype, ATG caused a significant induction of indoleamine 2,3-dioxygenase expression and a concomitant increase in levels of tryptophan metabolites in the supernatants of DC. Further, ATG-DC did not induce the proliferation of allogeneic T cells in a mixed lymphocyte reaction but actively suppressed the T cell proliferation induced by mature DC. These data suggest that besides its well-known effect on T cells, ATG modulates the phenotype of DC in a tolerogenic way, which might constitute an essential part of its immunosuppressive action in vivo.

## 1. Introduction

Allogeneic hematopoietic stem cell transplantation (aHSCT) is one of the most effective treatments for hematologic malignancies. However, graft-versus-host disease (GvHD) is still the major cause of mortality in aHSCT [1].

Dendritic cells (DC) play a crucial role in the pathogenesis of GvHD. But aside from their ability to induce GvHD, DC can also exhibit tolerogenic properties that may be exploited in the prevention and treatment of GvHD [1]. Tolerogenic DC are characterized by a semi-mature phenotype with high expression of MHC II (major histocompatibility complex) and low expression of costimulatory B7 molecules. They mainly secrete immunosuppressive mediators, such as IL-10, and lack the production of proinflammatory cytokines such as IL-12 [2].

Immunosuppressive medication of patients undergoing aHSCT includes antithymocyte globulin (ATG)-Fresenius (now Grafalon^®^), a polyclonal antibody-mixture raised in rabbits against the human lymphoblastic T cell line Jurkat [3]. Administration of ATG leads to the depletion of T lymphocytes [4]. ATG also affects other immune cells, including dendritic cells (DC) [4], as it directly binds to different DC surface antigens such as CD1a, CD80, CD86, CD206, and HLA-DR [5,6]. Furthermore, ATG induces different signaling pathways in DC, e.g., the activation of the protein kinases ERK1/2 and p38, as well as NF-κB signaling [6], and depletes circulating DC [7]. However, since host-derived tissue-resident DC subsets persist over a prolonged period after aHSCT [8], the effects of ATG on DC probably exceed depletion. And indeed, ATG interferes with various aspects of DC function: it modulates DC differentiation, inhibits maturation induced by different stimuli, suppresses the IL-12 secretion of mature DC, and reduces the ability of mature DC to stimulate T cells. Furthermore, it seems to induce a tolerogenic phenotype accompanied by mRNA expression of indoleamine 2,3-dioxygenase (IDO) [5,6,9,10]. In patients with ATG prophylaxis, we previously reported systemic release of tryptophan metabolites on the day of aHSCT also indicating activation of IDO by ATG [11].

IDO catalyzes the initial step of the tryptophan metabolism [12]. Its expression is a characteristic of tolerogenic DC and is associated with enhanced tryptophan degradation [13]. Both the deprivation of tryptophan and the accumulation of tryptophan metabolites represent potent immune-regulatory mechanisms [14,15]. Additionally, IDO-expressing DC are able to induce regulatory T cells [14] and contribute to renal allograft tolerance in mice [16].

Since published studies on the effect of ATG on human DC were conducted with different ATG preparations and experimental settings [5,6,9,10], we chose ATG-Fresenius, which is approved for GvHD prophylaxis and had the strongest effect on the release of tryptophan metabolites in patients [11], and studied the effect of ATG on immature DC in the absence of any maturation stimulus. This setting reflects the clinical situation where patients receive ATG prior to aHSCT and tissue-resident immature DC encounter ATG. ATG induced a tolerogenic DC phenotype with increased expression of IDO and an increased tryptophan metabolism. In accordance with their tolerogenic phenotype, ATG-DC negatively influenced the T cell proliferation induced by LPS-matured DC. These results clearly demonstrate that ATG is a potent tolerogenic stimulus for DC. They also suggest that the mechanism underlying the clinical effects of ATG on graft rejection and GvHD might go far beyond T lymphocyte depletion.

## 2. Results

### 2.1. ATG Induces a Semi-Mature Phenotype in DC

**Figure 1:**
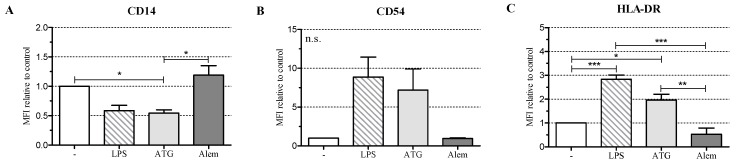

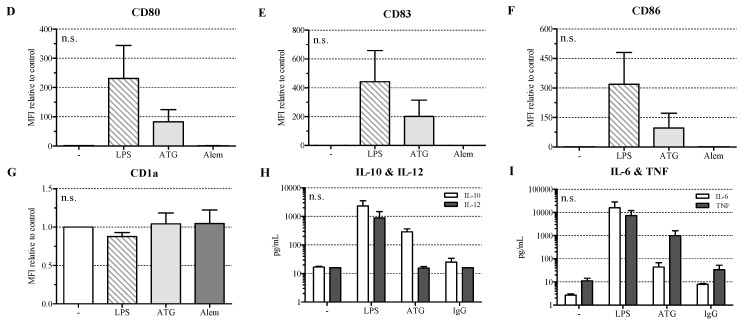
Antithymocyte globulin (ATG) induces a semi-mature dendritic cells (DC) phenotype. Monocyte-derived DC were stimulated with or without LPS (10 ng/mL), ATG (100 µg/mL), Alemtuzumab (Alem; 100 µg/mL) (**A**–**G**), or polyclonal rabbit IgG (100 µg/mL) (**H**,**I**); (**A**–**G**) After 48 h, cells were harvested, washed, and stained with fluorochrome-conjugated monoclonal antibodies against CD14 (**A**); CD54 (**B**); HLA-DR (**C**); CD80 (**D**); CD83 (**E**); CD86 (**F**) and CD1a (**G**). Samples were analyzed by flow cytometry. Shown is the mean of the median fluorescence intensity (MFI) (isotype subtracted) relative to unstimulated DC ± SEM (*n* = 3); (**H**,**I**) After 24 h, the supernatants were harvested and the amounts of IL-10, IL-12, IL-6, and TNF were determined by ELISA. Data are means ± SEM (*n* ≥ 3). Statistical analysis was performed with one-way ANOVA (* *p* ≤ 0.05, ** *p* ≤ 0.01, *** *p* ≤ 0.001) (n.s.: not significant).

To investigate the influence of ATG on the maturation of DC, we cultivated monocyte-derived DC with or without LPS (10 ng/mL) or with a therapeutically relevant concentration of ATG-Fresenius (100 µg/mL) (now Grafalon^®^) [3]. In addition, we incubated DC with Alemtuzumab (100 µg/mL), another immunosuppressive drug directed against lymphocytes that is approved for the prevention of GvHD [17]. After 48 h we analyzed the expression of CD1a, CD11c, CD14, CD54, DC-SIGN (CD209), HLA-DR, CD80, CD83, and CD86. LPS induced a fully mature DC phenotype with significantly increased expression of HLA-DR, low expression of CD14, and high expression of CD54, CD80, CD83, and CD86 (Figure 1A–F, Figure S2).

ATG induced a semi-mature DC phenotype characterized by significantly reduced CD14 and increased HLA-DR expression. Furthermore, ATG slightly increased CD80, CD83, CD86, and CD54 expression. Alemtuzumab did not affect the expression of the investigated surface markers. The expression of CD1a (Figure 1G and Figure S2), CD11c, and DC-SIGN (Figure S1A,B) was not changed by any of the applied stimuli.

To study whether stimulatory pathways are still functional in ATG-treated DC, we stimulated monocyte-derived DC either with ATG for 48 h or incubated them for the same time without stimulation. After 48 h, cells received LPS stimulation for another 48 h. Finally, DC surface markers were analyzed by means of flow cytometry (Figure S3). With the exception of CD83, which was slightly, but not significantly lower expressed in DC with preceding ATG-treatment, the levels of the other analyzed DC markers (HLA-DR, CD80, CD86, CD1a) were comparable between cells with and without ATG treatment.

### 2.2. ATG Induces IL-10 Secretion in DC

Next, we analyzed the effect of ATG on cytokine secretion by DC. Since ATG is a polyclonal preparation of rabbit IgG antibodies against human T lymphoblasts [3], we used normal polyclonal rabbit IgG as a control. LPS-DC produced large amounts of IL-10, IL-12, IL-6, and TNF. In contrast, ATG induced IL-10 but no IL-12, a typical feature of tolerogenic DC (Figure 1H,I). Furthermore, ATG-stimulated DC were only weak producers of IL-6 but secreted TNF. Neither ATG nor the IgG control influenced the investigated cytokines in monocytes, while LPS strongly induced their expression (Figure S4).

### 2.3. ATG Induces mRNA Expression of IDO in DC

**Figure 2:**
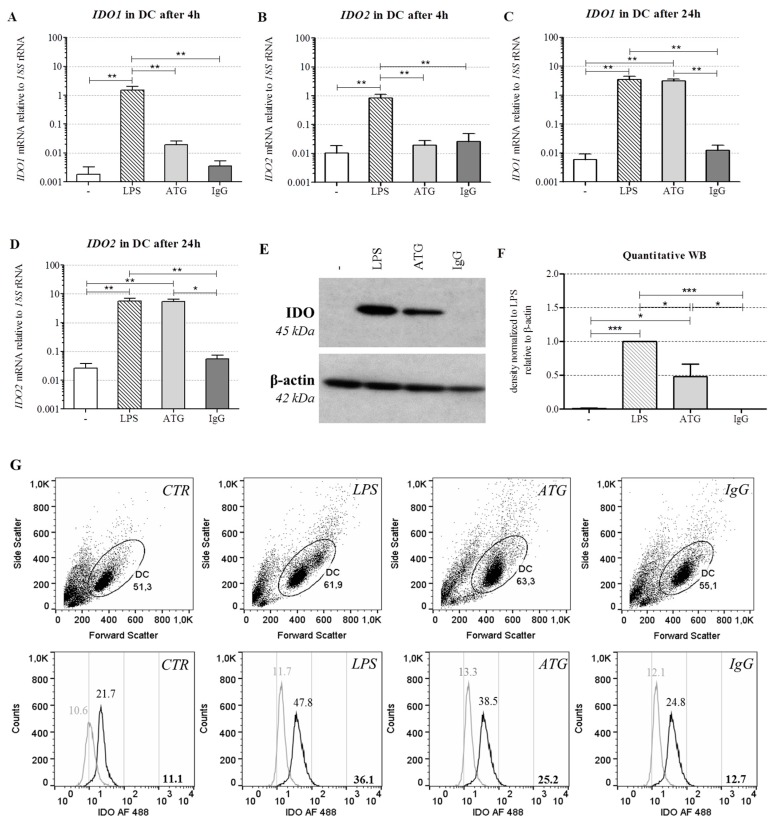
ATG induces IDO in DC at the mRNA and protein level. Monocyte-derived DC were cultivated with or without LPS (10 ng/mL), ATG (100 µg/mL), and IgG isotype control (100 µg/mL), respectively. (**A**–**D**) After 4 h and 24 h, mRNA expression of *IDO1* (**A**,**C**) and *IDO2* (**B**,**D**) was analyzed by means of quantitative real-time PCR relative to *18S* rRNA expression. Data are means ± SEM (*n* ≥ 3); (**E**) After 48 h, Western blot analysis was performed using antibodies against IDO and β-actin. Shown is one representative out of five independent experiments; (**F**) Densitometric analysis of five different experiments (normalized to the LPS signal). Data are means ± SEM; (**G**) Cells were harvested after 48 h, washed, and stained with Alexa Fluor (AF) 488-conjugated monoclonal anti-IDO antibody and the suitable isotype. Shown is a dot blot of the respective cell populations and overlaid histograms of the isotype (grey) and the anti-IDO antibody (black) of one representative of six experiments. Numbers indicate the median fluorescence of IDO, bold numbers in the lower right corner indicate the difference median fluorescence IDO—median fluorescence isotype. Statistical analysis was performed using one-way ANOVA (* *p* ≤ 0.05, ** *p* ≤ 0.01, *** *p* ≤ 0.001).

In order to investigate whether the tolerogenic phenotype of ATG-DC is accompanied by the expression of IDO, we analyzed mRNA expression of *IDO1* and *IDO2* after incubation with or without LPS (10 ng/mL), ATG (100 µg/mL), or the corresponding IgG control (100 µg/mL). After 4 h of incubation, LPS significantly enhanced the expression of *IDO1* and *IDO2* (Figure 2A,B).

After 24 h, both LPS and ATG strongly increased the expression of *IDO1* and *IDO2* (Figure 2C,D). In contrast to DC, ATG exerted no significant impact on *IDO1* or *IDO2* expression in monocytes (Figure S5A,B). Besides *IDO*, ATG and LPS also significantly increased the mRNA expression of the gene *KYNU* coding for kynureninase, another enzyme of the kynurenine pathway (Figure S5C). Since kynurenine is discussed as a ligand of the aryl hydrocarbon receptor (AHR) [18], we also investigated expression of this receptor. DC constitutively expressed *AHR* mRNA, but neither ATG nor LPS affected its expression (data not shown).

### 2.4. ATG Induces IDO at the Protein Level

To quantify IDO at the protein level, monocyte-derived DC were cultured for 48 h with or without LPS (10 ng/mL), ATG (100 µg/mL), or the IgG isotype control (100 µg/mL). In accordance with the mRNA expression data, Western blot analysis revealed a clear induction of IDO protein expression in DC by ATG (Figure 2E,F). Furthermore, we performed intracellular staining of IDO in DC and confirmed the Western blot results by means of flow cytometry (Figure 2G). In line with the mRNA data, ATG did not affect IDO expression in monocytes (data not shown).

### 2.5. ATG Stimulates Tryptophan Metabolism

**Figure 3:**
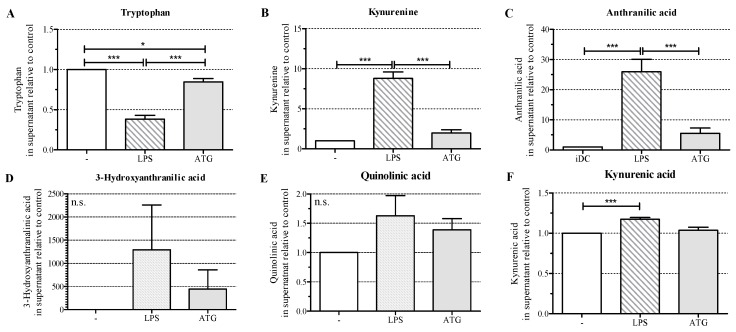
ATG enhances the tryptophan metabolism. Monocyte-derived DC were incubated for 48 h with or without LPS (10 ng/mL) or ATG (100 µg/mL). After 48 h, supernatants were harvested and levels of tryptophan (**A**); kynurenine (**B**); anthranilic acid (**C**); 3-hydroxyanthranilic acid (**D**); quinolinic acid (**E**) and kynurenic acid (**F**) were measured by means of liquid chromatography-electrospray ionization-tandem mass spectrometry (LC-ESI-MS/MS). Data are means ± SEM (*n* = 6) of the concentration of the respective metabolites (µM) relative to the unstimulated control. Statistical analysis was performed with one-way ANOVA (* *p* ≤ 0.05, *** *p* ≤ 0.001) (n.s.: not significant).

To test whether the increased expression of IDO in response to ATG affected the production of kynurenines, we cultured monocyte-derived DC with or without LPS (10 ng/mL) or ATG (100 µg/mL) for 48 h and analyzed the levels of tryptophan and selected kynurenines in DC culture supernatants by means of LC-MS/MS. We detected significantly decreased tryptophan levels in the supernatants of DC stimulated with LPS or ATG, indicating that increased IDO expression indeed led to enhanced degradation of tryptophan (Figure 3A).

Furthermore, LPS significantly increased the levels of the kynurenine pathway intermediates kynurenine, anthranilic acid, and kynurenic acid, but not the levels of 3-hydroxyanthranilic acid and quinolinic acid (Figure 3B–F). ATG slightly enhanced the levels of the analyzed kynurenine pathway intermediates.

### 2.6. ATG-Stimulated DC Suppress the Proliferation of Allogeneic T Cells

**Figure 4:**
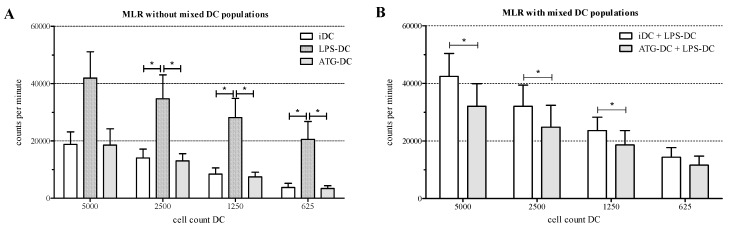
Reduced proliferation of allogeneic T cells in the presence of ATG-DC. Monocyte derived-DC were stimulated for 48 h with or without LPS (10 ng/mL) or ATG (100 µg/mL). Cells were harvested and washed. (**A**) Different numbers of DC (5000, 2500, 1250, 625) were cocultured with allogeneic T lymphocytes in the absence of ATG or LPS; (**B**) LPS-matured DC were mixed 1:1 either with immature DC (iDC) or ATG-DC. Different numbers (5000, 2500, 1250, 625) of mixed DC were cocultured with allogeneic T lymphocytes in the absence of ATG or LPS; (**A**,**B**) After five days, the proliferation rate of T cells was quantified by means of [^3^H]-thymidine assay. Data are means ± SEM (*n* = 4). Statistical analysis was performed using one-way ANOVA (**A**) or Student’s *t*-test (**B**) (* *p* ≤ 0.05).

To determine the T cell stimulatory capacity of ATG-stimulated DC, we incubated monocyte-derived DC with or without LPS (10 ng/mL), ATG (100 µg/mL), and IgG isotype control, respectively, for 48 h. Next, we co-cultured different numbers of these DC for 5 days with allogeneic T cells in the absence of LPS, ATG, or IgG control, and analyzed proliferation of the co-cultures by means of a [^3^H]-thymidine proliferation assay. LPS-DC strongly induced proliferation of T lymphocytes. However, neither ATG-DC (Figure 4A) nor IgG control (data not shown) induced T cell proliferation.

Finally, we mixed LPS-matured DC with ATG-DC or immature DC, respectively, at a ratio of 1:1, and co-cultured the mixtures with allogeneic T lymphocytes in the absence of LPS or ATG. In this setting, a reduced proliferation of allogeneic T cells was observed (Figure 4B and Figure S6). This effect was significant for three out of four DC concentrations. The IgG isotype control had no effect on the proliferation of T cells (data not shown).

To study the possible mechanisms underlying the interaction of ATG-DC with T cells, we analyzed the expression of T cell-inhibitory molecules on the surface of ATG-DC. We detected a lower level of PD-L1 expression on ATG-DC compared to LPS-DC, and a higher level of TIM-3 expression on ATG-DC compared to LPS-DC (Figure S7).

## 3. Discussion

There is broad consensus that the depletion of T cells is mainly responsible for the immunosuppressive effect of ATG [4]. ATG is also known to induce regulatory T cells [19]. Furthermore, ATG has been reported to bind to neutrophils, monocytes, and DC, to interact with various surface antigens, and to influence the biology of DC [5,6,9,10,20]. However, these studies used different ATG preparations, each with a unique polyclonal mixture of IgG antibodies [4], with varying binding capacities [5,20]. Furthermore, the experimental settings differed. Since we aimed to mimic the encounter of immature (tissue-resident) DC with ATG, we chose the incubation of immature DC with ATG-Fresenius as our experimental setting.

As Naujokat et al. [6] had detected the induction of apoptosis in immature and mature DC by 10 and 100 µg/mL ATG-Fresenius, respectively, we determined cell count in our iDC cultures in response to 2 days of incubation with 100 µg/mL ATG, a concentration reached in vivo in patients [3], and detected no difference compared to the control without ATG (data not shown).

ATG generated DC with significantly reduced expression of CD14, enhanced expression of HLA-DR, and slightly increased expression of CD54, CD80, CD83, and CD86. This implies that ATG does not induce a fully mature, but rather a semi-mature DC phenotype, which is a typical feature of tolerogenic DC [2]. Our data are in line with data from Leitner et al. [5], who detected increased expression of CD83 and CD86 in response to incubation of immature DC with ATG-Fresenius. Gillet-Hladky et al. [10] also analyzed the surface antigen expression of DC in response to ATG. However, they used a different ATG preparation and incubated the cells simultaneously with ATG and LPS. Therefore, their results cannot be compared.

In further experiments, we tested the ability of ATG-DC to react to subsequent LPS stimulation and detected no differences in the expression of surface markers compared to DC stimulated only with LPS. This indicates that the phenotype induced by ATG is reversible and that ATG-DC maintain their reactivity towards TLR4 ligands.

In line with the semi-mature phenotype, we found ATG-DC to produce IL-10, and no IL-12. Further, ATG-DC produced only a small quantity of IL-6. In contrast to these findings, TNF production was clearly detectable in the presence of ATG. This seems to be contradictory to a tolerogenic phenotype since TNF is widely known as a pro-inflammatory cytokine. However, TNF has also been used to expand human regulatory T cells and to fortify their suppressive capacity in vitro [21]. The high content of TNF in the serum of mice suffering from acute GvHD has consistently been demonstrated to selectively activate regulatory T cells [22]. Since the application of ATG is accompanied by the induction of regulatory T cells with strong suppressive capacity in vitro and in vivo [23,24], the TNF secretion by our ATG-DC might support this enrichment of regulatory T cells after ATG administration in patients.

The effect of ATG on cytokine secretion was specific for DC, because we could not detect any effect on cytokine secretion by monocytes. In contrast to our results, Leitner et al. [5] reported immature DC to secrete TNF, but not IL-10, IL-1β, and IL-6 in response to incubation with ATG. However, since these authors did not show any data on TNF and IL-6, one cannot tell whether the amounts of TNF and IL-6 secreted by their ATG-DC are in the range of the amounts secreted by our ATG-DC. Since they detected about 500 times less IL-10 in the supernatant of LPS-stimulated DC than we did, one has to consider the possibility that the different results obtained for IL-10 secretion might be due to technical rather than biological reasons.

The expression of IDO, which catalyzes the initial and rate-limiting step of the kynurenine pathway, is known to mediate the immunosuppressive effect of tolerogenic DC, such as inhibiting T cell functions, activating regulatory T cells, and suppressing natural killer cells [13]. In our hands, ATG induced the expression of IDO at both the mRNA and the protein level. Our data are in accordance with Leitner et al. [5], who detected increased *IDO* mRNA expression after incubation of iDC with ATG. Interestingly, Gillet-Hladky et al. [10], who tested the influence of a different ATG preparation on the differentiation of DC, also found increased mRNA expression of *IDO* in DC. This indicates that the effects of ATG on IDO are not restricted to a certain type of ATG preparation or phase of DC development.

In concordance with the expression of IDO, we detected enhanced depletion of tryptophan and slightly increased levels of kynurenine pathway intermediates in the culture supernatants of ATG-DC. Both the formation of these metabolites and the depletion of tryptophan are known to have immunoregulatory effects [12]. In mice, kynurenine has been identified as a ligand of the aryl hydrocarbon receptor (AHR) [18] and the interaction of kynurenine with AHR has been demonstrated to induce the expression of IDO [25]. Since we detected constitutive *AHR* mRNA expression in DC, the interaction of kynurenine with AHR might represent a feedback loop fortifying the expression of IDO in the presence of ATG.

Interestingly, our previous analysis of urinary levels of tryptophan metabolites in patients undergoing aHSCT revealed marked increases in the levels of kynurenine and quinolinic acid in response to the administration of ATG [11]. Together with the findings of the present publication, these data strongly suggest that the influence of ATG on tryptophan metabolism in DC (and possibly other cell types) is responsible for the ATG-induced rise in tryptophan metabolites in the urine of aHSCT patients.

ATG-DC did not induce the proliferation of allogeneic T cells. Similar results were obtained by Monti et al. [9] who reported that the preincubation of immature DC with ATG has no effect on their capacity to stimulate allogeneic T cell proliferation. However, since they used a different ATG preparation, the comparability of the results is limited. Naujokat et al. also investigated the role of ATG-Fresenius in an allogeneic MLR, but they tested its effect on proliferation induced by mature DC, an experimental setting different from ours [6]. However, our ATG-DC were not only unable to activate allogeneic T cells but when we mixed them with potent stimulatory LPS-matured DC, ATG-DC suppressed the proliferation of allogeneic T cells.

Tolerogenic DC can limit T cell proliferation through various mechanisms. Low expression of (co-)stimulatory molecules, low IL-12 and high IL-10 production, and/or high IDO expression can induce regulatory T cells which in turn suppress proliferation [2]. ATG-DC have these characteristics, but they suppress proliferation in a co-culture with potent LPS-DCs which express high levels of co-stimulatory molecules, secrete high levels of IL-12, and express higher IDO levels as compared to ATG-DCs. Therefore, it is unlikely that the above mentioned factors are responsible for the limited proliferation in our experimental setting. More likely ATG-DC directly inhibit T cell proliferation, e.g., via expression of inhibitory receptors such as PD-L1 or TIM-3. Indeed, we detected a higher level of TIM-3 expression on ATG-DC compared to LPS-DC. Besides its direct effects on DC [26], expression of TIM-3 on lymphoma endothelial cells has been shown to suppress the activation of CD4+ T cells [27]. Therefore, the TIM-3 expression on ATG-DC might represent a mechanism for a direct negative interaction between DC and T cells.

## 4. Material and Methods

### 4.1. Chemicals

Unless otherwise indicated, chemicals were purchased from Sigma-Aldrich (Munich, Germany).

### 4.2. Isolation of Monocytes and T Lymphocytes

With approval from the local ethics committee, monocytes and T lymphocytes were isolated from healthy donors as described previously [28].

### 4.3. Culture of Monocytes and Monocyte-Derived DC

Monocytes were cultured at a density of 0.5 to 1.0 × 10^6^/mL in RPMI-1640 (Gibco, Karlsruhe, Germany) supplemented with 2% human AB serum (PAN Biotech, Aidenbach, Germany), l-glutamine (2 mmol/L), penicillin (50 U/mL), and streptomycin (50 mg/mL; all from Life Technologies, Karlsruhe, Germany). For differentiation into DC, 0.5 to 1.0 × 10^6^ monocytes/mL were cultured for six days in medium as described above, supplemented with 10% fetal calf serum (PAN Biotech), IL-4 (144 U/mL), and granulocyte macrophage colony-stimulating factor (GM-CSF, 225 U/mL; both from PeproTech, Hamburg, Germany). Monocytes and DC were incubated for 4 h, 24 h, and 48 h, respectively, with or without LPS (from *Salmonella abortus equi* S-form, Enzo Life Sciences, Lörrach, Germany), ATG (Fresenius, Bad Homburg, Germany) (now named Grafalon^®^, distributed by Neovii Biotech, Gräfelfing, Germany) (100 µg/mL), IgG isotype control (polyclonal, rabbit, Molecular Innovations, Novi, MI, USA) (100 µg/mL) or Alemtuzumab (monoclonal, human, anti-CD52, Genzyme, Cambridge, MA, USA) (100 µg/mL).

### 4.4. Determination of Cytokines

Determination of cytokines in culture supernatants was performed using ELISA kits from R&D Systems (Minneapolis, MN, USA).

### 4.5. Preparation of RNA, Reverse Transcription, and Quantitative Real-Time PCR

Total cellular RNA was extracted using the RNeasy Mini Kit (Qiagen, Hilden, Germany). RNA concentration was measured using a NanoDrop Spectrophotometer (Thermo Fisher Scientific, Schwerte, Germany). Reverse transcription was performed as described previously [29]. Primer sequences (all from Eurofins MWG Operon, Ebersberg, Germany) are provided in Table S1.

### 4.6. Preparation of Whole Cell Lysates and Western Blotting

Whole cell lysates were prepared using RIPA buffer (Sigma-Aldrich) and quantified with DC Protein Assay (Bio-Rad Laboratories, Hercules, CA, USA). After blotting, proteins were detected using the antibodies listed in Table S2. Densitometric analyses were performed using the ChemiDoc MP Imaging System and Image Lab^TM^ software (both Bio-Rad Laboratories).

### 4.7. Analysis of Tryptophan Metabolites

Culture supernatants were stored at −20 °C until liquid chromatography-electrospray ionization-tandem mass spectrometry (LC-ESI-MS/MS) of tryptophan, kynurenine, anthranilic acid, 3-hydroxyanthranilic acid, quinolinic acid, and kynurenic acid, according to published protocols [30].

### 4.8. Flow Cytometry Analysis

Cells were washed twice with cold PBS (Gibco, Karlsruhe, Germany) containing 0.1% sodium azide and 0.6 mg/mL immunoglobulin, and were stained with the monoclonal antibodies listed in Table S3. For intracellular staining, the Fixation & Permeabilization Buffer Kit was used (R&D Systems). Data were recorded on a FACS Calibur cytometer and analyzed using CellQuestPro (both BD, Heidelberg, Germany) and FlowJo (Tree Star, Ashland, OR, USA).

### 4.9. Mixed Lymphocyte Reaction (MLR) and ^3^H-Thymidine Assay

Different numbers of DC were cocultured with 100,000 allogeneic T lymphocytes in RPMI containing 5% AB serum, l-glutamine (2 mmol/L), penicillin (50 U/mL), and streptomycin (50 mg/mL; all from Life Technologies). On day 5, 0.5 µCi/0.2 mL [^3^H]-thymidine (Amersham Pharmacia, Piscataway, NJ, USA) was added. Incorporated radioactivity was quantified after 24 h by means of a beta counter (Perkin Elmer, Gaithersburg, MD, USA).

### 4.10. Statistical Analysis

Statistical analysis was performed with one-way ANOVA or Student’s *t*-test using Graph Pad Prism 5 software (GraphPad Software, La Jolla, CA, USA). A value of *p* < 0.05 was considered statistically significant.

## 5. Conclusions

Our findings support the growing body of evidence on the effects of ATG on immune cells other than T cells, and clearly show that ATG-Fresenius induces a tolerogenic phenotype in human monocyte-derived DC that potently exerts its regulatory effects in an allogeneic in vitro-setting. Interestingly, recent work shows that ATG directly induces regulatory T cells in vivo [24]. The induction of tolerogenic DC by ATG might be a mechanism supporting the establishment of this T cell population, which is beneficial in the context of aHSCT [31]. The findings described in the current work are focused on the in vitro-analysis of an isolated cell population. However, ATG is known to affect various immune cell types in vivo [4]. This complex interplay between different immune cell populations and ATG should be carefully investigated in vivo in further studies.

In summary, our data together with published data suggest that the beneficial effect of ATG in transplantation patients is not only the result of its T cell-depleting activity but may also be caused by its tolerogenic “side effects”, namely direct induction of regulatory T cells and tolerogenic DC.

## Supplementary Materials

The following are available online at www.mdpi.com/1422-0067/17/12/2081/s1.
