# Supplementary material for: Antithymocyte Globulin Induces a Tolerogenic Phenotype in Human Dendritic Cells
Source: Int J Mol Sci. 2016 Dec 11;17(12):2081. doi: 10.3390/ijms17122081 (PMC5187881; doi:10.3390/ijms17122081)
Supplement: Supplementary file 1 [file ijms-17-02081-s001.pdf]

Supplementary Materials: Antithymocyte Globulin Induces a Tolerogenic Phenotype in Human Dendritic Cells

Tobias Roider, Michael Katzfuß, Carina Matos, Katrin Singer, Kathrin Renner, Peter J. Oefner, Katja Dettmer-Wilde, Wolfgang Herr, Ernst Holler, Marina Kreutz and Katrin Peter

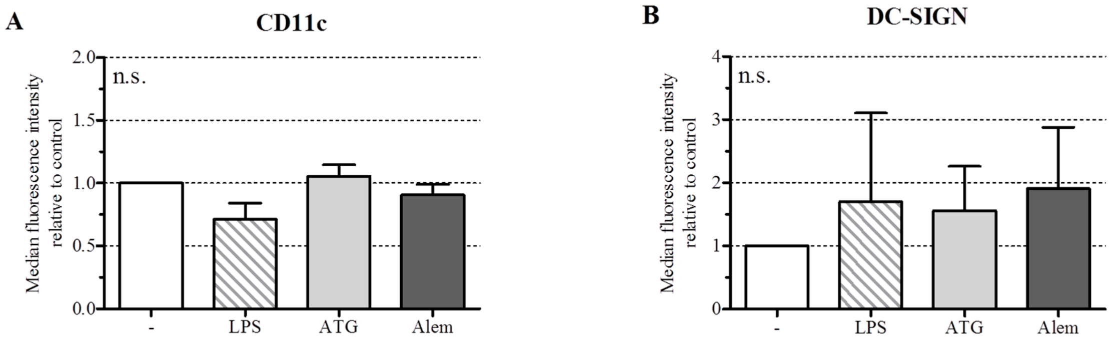

Figure S1. ATG does not affect surface expression of CD11c and DC-SIGN in DC. Monocyte-derived DC were stimulated with or without LPS (10 ng/mL), ATG (100 µg/mL) or Alemtuzumab (Alem; 100 µg/mL). After 48 h cells were harvested, washed and stained with fluorochrome-conjugated monoclonal antibodies against CD11c (**A**) and CD209 (DC-SIGN) (**B**). Samples were analyzed by flow cytometry. Shown is the mean of the median fluorescence intensity (MFI) (isotype subtracted) relative to unstimulated DC ± SEM (n = 3). Statistical analysis was performed with one-way ANOVA (n.s.: not significant).

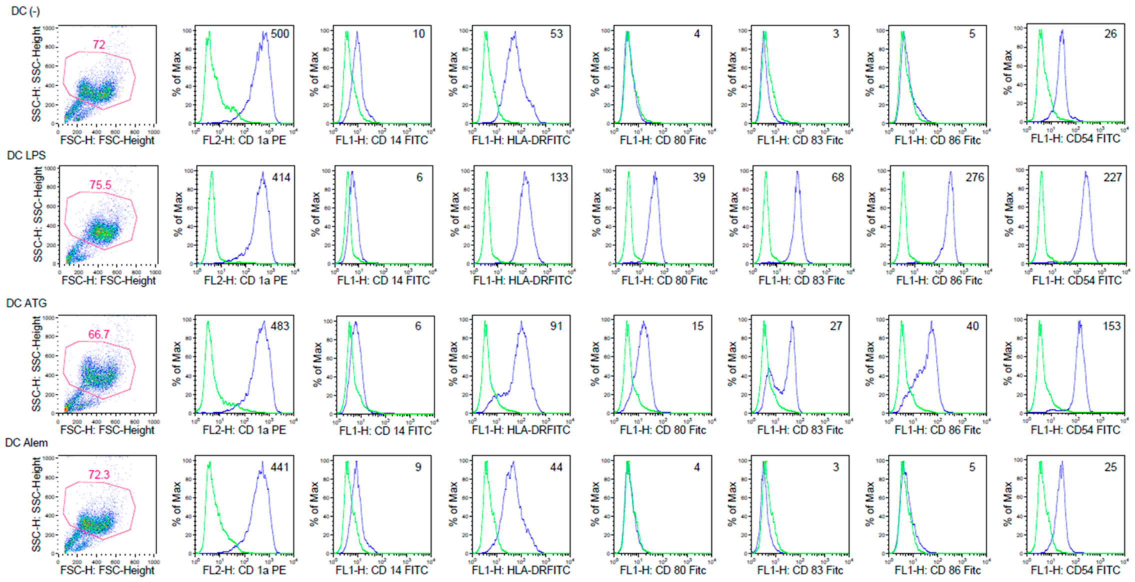

Figure S2. Influence of ATG, LPS and Alemtuzumab on surface antigen expression in DC (one representative experiment). Monocyte-derived DC were stimulated with or without LPS (10 ng/mL), ATG (100 µg/mL) or Alemtuzumab (Alem; 100 µg/mL). After 48 h cells were harvested, washed and stained with fluorochrome-conjugated monoclonal antibodies against CD1a, CD14, HLA-DR, CD80, CD83, CD86 and CD54. Samples were analyzed by flow cytometry. The median fluorescence intensity (MFI) (isotype subtracted) is given in the upper right corner of each blot (median fluorescence intensity for isotypes was always < 5.5). Shown is one representative experiment out of 3 independent experiments.

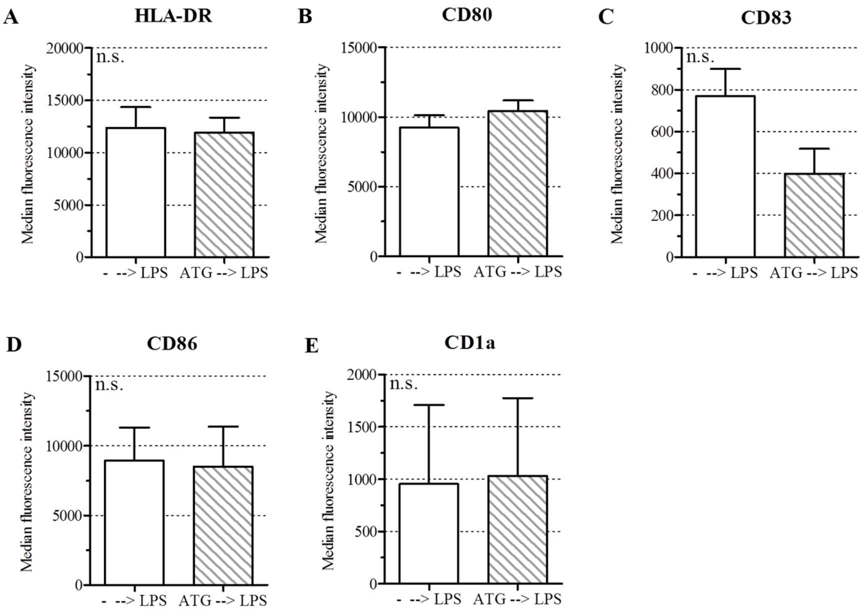

Figure S3. ATG-DC own the capability to upregulate surface antigen expression in response to stimulation with LPS. Monocyte-derived DC were stimulated with or without ATG (100 µg/mL) for 48 h. Subsequently, they received LPS-stimulation (10 ng/mL) for additional 48 h. Finally, cells were harvested, washed and stained with fluorochrome-conjugated monoclonal antibodies against HLA-DR (**A**); CD80 (**B**); CD83 (**C**); CD86 (**D**) and CD1a (**E**). Samples were analyzed by flow cytometry. Shown is the mean of the median fluorescence intensity (MFI) (isotype subtracted) of 3 (CD80 n = 2) independent experiments ± SEM. Statistical analysis was performed with Student's t-test (n.s.: not significant).

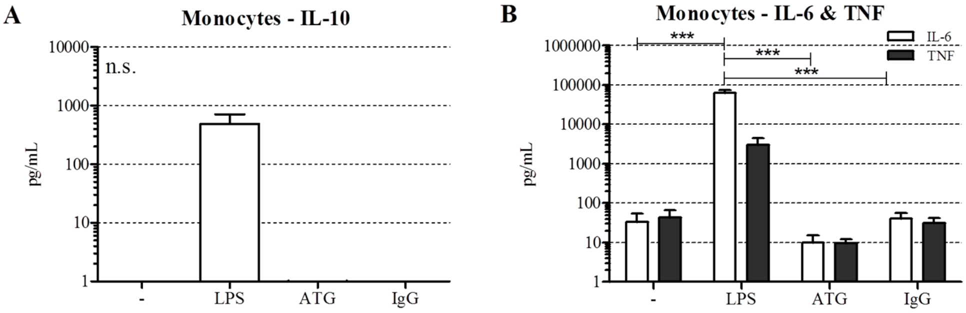

Figure S4. ATG does not influence cytokine secretion by human monocytes. Human freshly isolated monocytes were stimulated with or without LPS (100 ng/mL), ATG (100 µg/mL) or polyclonal rabbit IgG (100 µg/mL). After 24 h the supernatants were harvested and the concentrations of IL-10 (**A**), IL-6 (**B**), and TNF (**B**) were quantified by ELISA. Data are means ± SEM ($n \geq 3$). Statistical analysis was performed with one-way ANOVA (*** $p \leq 0.001$) (n.s.: not significant).

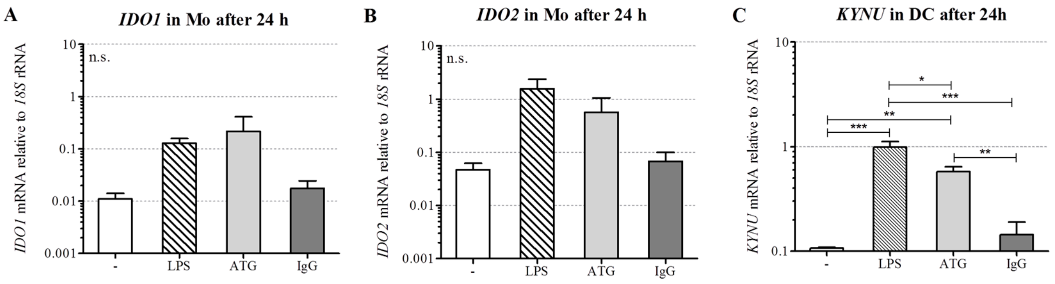

Figure S5. ATG induces *IDO* mRNA expression in monocytes and *KYNU* mRNA expression in DC. (**A**,**B**) Freshly isolated human monocytes (Mo) were incubated for 24 h with or without LPS (100 ng/mL), ATG (100 µg/mL) or polyclonal rabbit IgG isotype control (100 µg/mL); (**C**) Monocyte-derived DC were incubated for 24 h with or without LPS (10 ng/mL), ATG (100 µg/mL) or IgG isotype control (100 µg/mL). After isolation of total RNA, the mRNA expression of *IDO1* (**A**); *IDO2* (**B**) or *KYNU* (**C**) was analyzed by quantitative real-time PCR relative to *18S* rRNA expression. Data are means ± SEM ((**A**,**B**) $n = 3$; (**C**) $n = 4$). Statistical analysis was performed with one-way ANOVA (* $p \leq 0.05$, ** $p \leq 0.01$, *** $p \leq 0.001$) (n.s.: not significant).

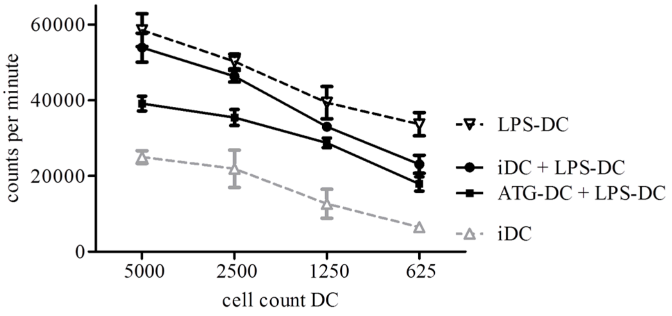

Compared samples	5000 DC	2500 DC	1250 DC	625 DC
ATG-DC + LPS-DC vs iDC + LPS-DC	*	n.s.	n.s.	n.s.
ATG-DC + LPS-DC vs iDC	n.s.	*	*	*
ATG-DC + LPS-DC vs LPS-DC	*	*	ns	**
iDC + LPS-DC vs iDC	***	**	**	**
iDC + LPS-DC vs LPS-DC	n.s.	n.s.	n.s.	*
iDC vs LPS-DC	***	***	***	***

Figure S6. Reduced proliferation of allogeneic T cells in the presence of ATG-DC (one representative experiment). Monocyte-derived DC were stimulated for 48 h with or without LPS (10 ng/mL) or ATG (100 µg/mL). Cells were harvested and washed. LPS-matured DC were mixed 1:1 either with immature DC (iDC) or ATG-DC. As controls pure iDC or mDC populations were used. Different numbers (5000, 2500, 1250, 625) of pure or mixed DC were cocultured with allogeneic T lymphocytes in the absence of ATG or LPS. All samples were pipetted in triplicates. After five days the proliferation rate of T cells was analyzed by means of [^{3}H]-thymidine assay. Data represent the mean ± SEM of the analyzed triplicates of one representative experiment. Statistical analysis (see table below figure) was performed using one-way ANOVA (* $p \leq 0.05$, ** $p \leq 0.01$, *** $p \leq 0.001$) (n.s.: not significant).

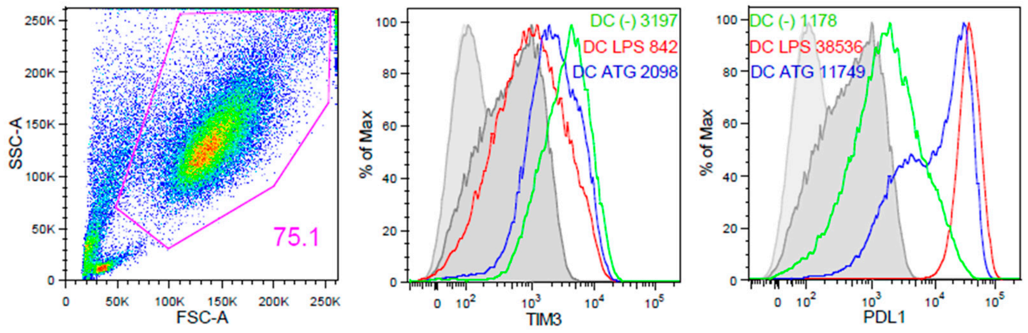

Figure S7. Influence of ATG or LPS on inhibitory surface antigen expression in DC (one representative experiment). Monocyte-derived DC were stimulated with or without LPS (10 ng/mL) or ATG (100 µg/mL). After 48 h cells were harvested, washed and stained with fluorochrome-conjugated monoclonal antibodies against TIM-3 and PD-L1. Samples were analyzed by flow cytometry. The median fluorescence intensity (MFI) (isotype subtracted) for TIM-3 and PD-L1 is given in the respective blots. Shown is one representative experiment out of 3 independent experiments.

Table S1. Oligonucleotide primers used for RT-qPCR.

Gene	Primer Sequence (5′ → 3′) (S, Sense; AS, Antisense)
18S rRNA	S: ACCGATTGGATGGTTTAGTGAG<br>AS: CCTACGGAAACCTTGTTACGAC
IDO1	S: GGTCATGGAGATGTCCGTAAGGT<br>AS: CCAGTTTCTTGGAGAGTTGGCAG
IDO2	S: GCATTCGTCATAGCAAGGAAAGTGG<br>AS: CTGTCAGCAAGTGGTCCTGTC
KYNU	S: TGTAGGAGCCAATGAGAAAGAAATAGCCC<br>AS: CGCATACTTTCTTCAATGTTAAGTCCGTG

Table S2. Primary and secondary antibodies used for western blotting.

Specificity	Manufacturer	Dilution
IDO	R&D Systems, Minneapolis, MN, USA	1:500
β-actin	Abcam, Cambridge, UK	1:1000
anti-goat, HRP-conjugated [a]	Dako, Glostrup, Denmark	1:2500
anti-mouse, HRP-conjugated [a]	Dako, Glostrup, Denmark	1:2500

[a] HRP: horseradish peroxidase.

Table S3. Antibodies used for flow cytometry.

Specificity	Conjugation	Manufacturer
CD1a	PE	Beckmann Coulter, Brea, CA, USA
CD14	FITC	Beckmann Coulter, Brea, CA, USA
CD83	FITC	Beckmann Coulter, Brea, CA, USA
HLA-DR	FITC	Beckmann Coulter, Brea, CA, USA
CD11c	FITC	eBioscience, San Diego, CA, USA
CD54	FITC	Cymbus Biotechnologies, Southampton, UK
DC-SIGN	PE	R&D Systems, Minneapolis, MN, USA
CD80	FITC	BD Biosciences, Franklin Lakes, NJ, USA
CD86	FITC	BD Biosciences, Franklin Lakes, NJ, USA
TIM-3	PE-Cy7	Biolegend, San Diego, CA, USA
PD-L1	PE-Cy7	Biolegend, San Diego, CA, USA
IDO	AF 488	R&D Systems, Minneapolis, MN, USA
Iso IDO	AF 488	R&D Systems, Minneapolis, MN, USA

Suitable isotypes (all from Beckmann Coulter, Brea, CA, USA) were used as controls.
